# Comprehensive management of advanced lymphoepithelioma-like hepatocellular carcinoma with immune-related colitis: a case report and literature review

**DOI:** 10.3389/fonc.2026.1763411

**Published:** 2026-03-12

**Authors:** Jizhe Zhang, Xianwen Zhang, Tingting Chen, Changren Zhu

**Affiliations:** 1 Department of Oncology, Northern Jiangsu People’s Hospital, Yangzhou, China; 2 Department of Pathology, Northern Jiangsu People's Hospital, Yangzhou, China

**Keywords:** case report, immune-related adverse events, immunotherapy, lymphoepithelioma-like hepatocellular carcinoma, multimodal therapy

## Abstract

Lymphoepithelioma-like hepatocellular carcinoma (LEL-HCC) is an exceedingly rare hepatocellular carcinoma subtype characterized by prominent lymphocyte infiltration. LEL-HCC accounts for fewer than 1% of all HCCs, and most patients present with resectable disease at initial diagnosis. For unresectable LEL-HCC, chemoimmunotherapy is employed based on the experience of the attending physician. However, no recognized effective treatment for advanced LEL-HCC has been established. We present the case of a 68-year-old male with unresectable advanced LEL-HCC, which was ultimately diagnosed by laparoscopic partial hepatectomy biopsy after intraoperative needle biopsy proved inconclusive. We chose a therapeutic regimen consisting of micellar paclitaxel plus cisplatin chemotherapy in combination with immunotherapy for the patient. After 5 months of chemoimmunotherapy, the patient achieved partial response (PR) that was sustained for 8 months until the latest follow-up. Notably, grade 3 immune-related colitis occurred 6 months after commencing immune checkpoint inhibitor (ICI) treatment, which was effectively managed with corticosteroids and Infliximab. This report presents the first documented severe immune-related adverse event (irAE) in a patient with LEL-HCC, thereby informing the comprehensive management of this rare malignancy.

## 1 Introduction

Lymphoepithelioma-like hepatocellular carcinoma (LEL-HCC) is a rare subtype of hepatocellular carcinoma characterized by prominent lymphocytic infiltration, distinct histologic features (1). LEL-HCC accounts for fewer than 1% of all HCC, and most patients present with resectable disease at initial diagnosis (2–4).The management of unresectable LEL-HCC typically involves comprehensive treatment modalities such as chemotherapy, immunotherapy, and radiotherapy. Due to the limited case volume of unresectable advanced LEL-HCC, no standardized treatment guidelines have been established, thereby rendering therapeutic strategies heavily dependent on the empirical judgment of the attending clinician. Furthermore, there have been no reported cases of immune-related adverse events in patients with LEL-HCC.

We present a 68-year-old male with unresectable advanced LEL-HCC, which was ultimately diagnosed via laparoscopic partial hepatectomy biopsy after intraoperative needle biopsy proved inconclusive. The patient achieved PR after 5 months of combined chemoimmunotherapy and radiotherapy for metastatic cervical lymph nodes. Notably, grade 3 immune-related colitis occurred 6 months after ICI initiation which resolved significantly with systemic corticosteroid treatment and Infliximab.

This case provides valuable insights into the multidisciplinary management of LEL-HCC and represents the first documentation of a severe irAE in LEL- HCC, thereby offering critical experience for its clinical management. Furthermore, this article comprehensively reviews recent advances in LEL-HCC, summarizing contemporary understanding of its epidemiology, pathogenesis, pathological features, treatment strategies, and prognostic factors. Collectively, our findings underscore the imperative for larger-scale clinical studies and systematic molecular analyses to elucidate the underlying mechanisms of LEL-HCC and establish evidence-based management guidelines.

## 2 Case description

**Figure 1:**
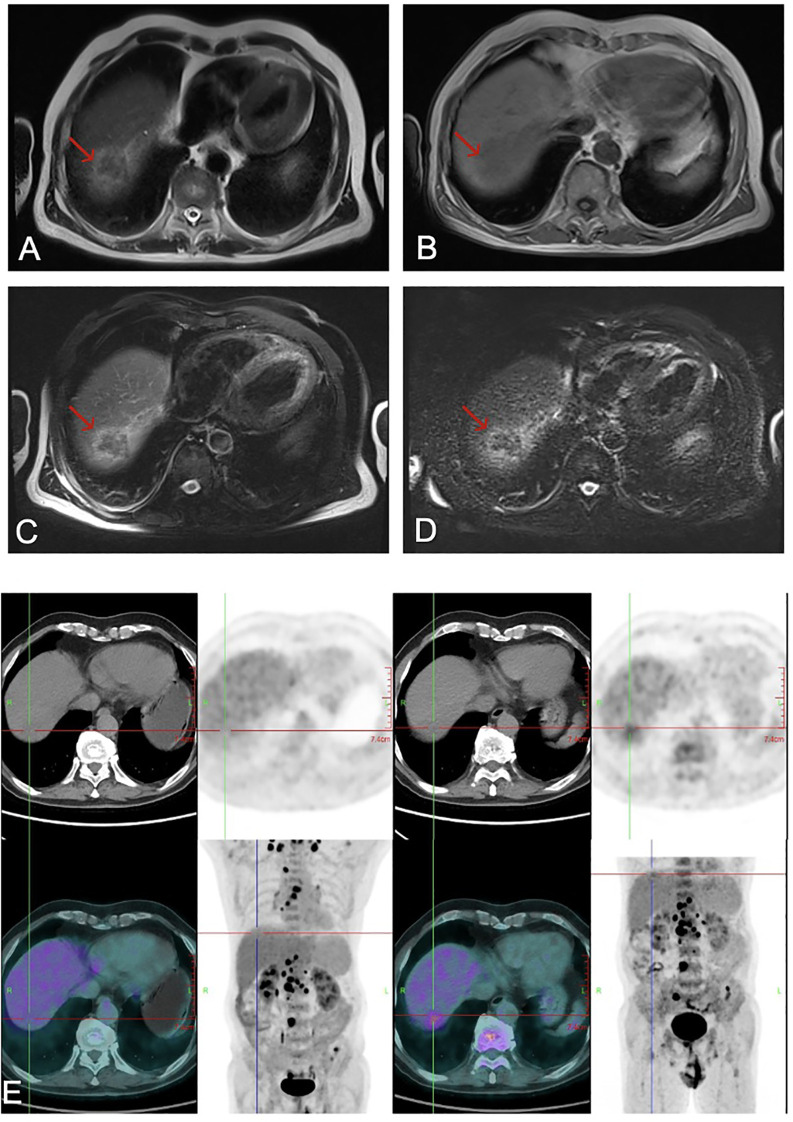
Abdominal MRI revealed a tumor (red arrow) in the right liver: **(A)** T2-weighted image; **(B)** T1-weighted image; **(C)** T2-weighted fat-suppressed image; and **(D)** diffusion-weighted image (DWI); **(E)** 18F-FDG PET-CT demonstrated widespread hypermetabolic foci throughout the body, consistent with disseminated metastatic disease. 18F-FDG, 18F-fluorodeoxyglucose; PET-CT, positron emission tomography–computed tomography; MRI, magnetic resonance imaging.

A 68-year-old male presented with a newly detected 34mm × 30mm enhancing mass in hepatic segment VI, identified on abdominal contrast-enhanced magnetic resonance Imaging (MRI). Hepatitis B virus (HBV) serology was notable for an isolated positive anti-HBc, while hepatitis C virus (HCV) serology was negative. Laboratory investigations revealed significantly elevated tumor markers: alpha-fetoprotein (AFP) at 757.8 ng/mL and carcinoembryonic antigen (CEA) at 26.47 ng/mL (reference ranges: 0–7 and 0–5 ng/mL, respectively). Cytokine profiling revealed an elevated interleukin-6 (IL-6) level of 77.68 pg/mL and a normal IL-5 level. Additionally, the anti-Ro52 antibody was positive. 18F-fluorodeoxyglucose positron emission tomography–computed tomography (18F-FDG PET/CT) imaging identified a hepatic lesion in the right posterior lobe with increased FDG uptake (55.1 × 27.4 mm; SUVmax 3.31). The scan demonstrated widespread metastases, including mediastinal and retroperitoneal lymph node involvement (SUVmax 8.45) and osteolytic metastases in the L4 vertebra (**Figure 1**).

To establish a definitive diagnosis, the patient underwent laparoscopic ultrasound-guided liver needle biopsy for intraoperative frozen section analysis. During the laparoscopic procedure, intraoperative exploration revealed no obvious macroscopic mass on the liver surface. Therefore, the surgeon relied on intraoperative ultrasound to precisely localize the suspicious area and performed an ultrasound-guided liver needle biopsy. A total of six tissue strips across two separate attempts were obtained. However, both intraoperative frozen section examinations returned results indicating no tumor tissue identified, with only lymphocytes present. Faced with the dilemma of an inability to establish a definitive diagnosis despite two intraoperative frozen section examinations following needle biopsies, obtaining sufficient and representative tissue samples to complete comprehensive pathological evaluation and etiological analysis became the only means to confirm the diagnosis and guide subsequent precision treatment. After urgent communication and joint assessment with the attending pathologist, the surgeon decided to perform a diagnostic partial hepatectomy to obtain adequate and definitive biopsy specimens. This procedure was performed laparoscopically, strictly adhering to aseptic and no-tumor principles. Histopathological examination revealed extensive necrosis with collagen fiber formation, surrounded by degenerated hepatic parenchyma and significant inflammatory cell infiltration. Focal areas showed scattered atypical large cells intermixed with inflammatory infiltrates, consistent with the histological features of LEL-HCC.

**Figure 2:**
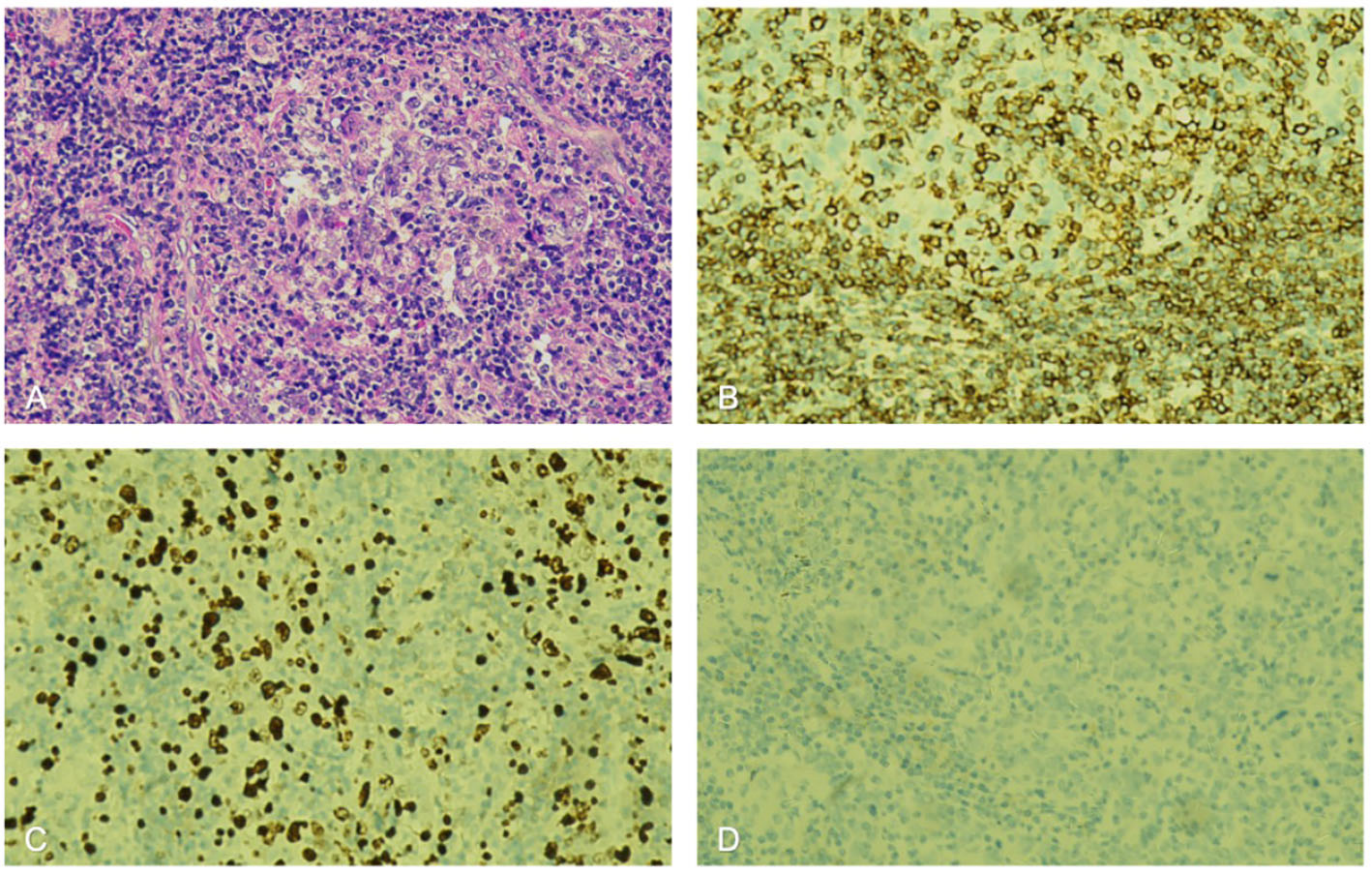
Pathological findings from the patient’s liver biopsy: **(A)** H&E staining; **(B)** Immunohistochemistry for LCA highlights dense lymphocytic infiltration; **(C)** Ki-67 staining shows a proliferative index of 50%; and **(D)** EBER *in situ* hybridization is negative. H&E, hematoxylin and eosin; LCA, leukocyte common antigen; EBER, Epstein-Barr virus-encoded RNA.

Further immunohistochemical (IHC) studies revealed dense lymphocytic infiltration highlighted by leukocyte common antigen (LCA) and a high Ki-67 proliferation index of 50%, indicating highly proliferative activity. Additionally, the tumor cells were negative for alpha-fetoprotein (AFP), glypican-3 (GPC3), hepatocyte antigen (HepPar-1), arginase-1 (Arg-1), cytokeratin 7 (CK7), CK19,CK5 and cluster of differentiation 68 (CD68). Immunohistochemical staining revealed that the tumor cells were positive for heat shock protein 70 (HSp70, cytoplasmic pattern), cytokeratin 5/6 (CAM5.2, focal), pan-cytokeratin (CKpan, focal), cytokeratin 20 (CK20, diffuse), epithelial membrane antigen (EMA, dot-like positivity), vimentin, SWI/SNF-related matrix-associated actin-dependent regulator of chromatin subfamily A member 4 (SMARCA4), and caudal-type homeobox 2 (CDX-2, focal). Integrase interactor 1 (INI1) showed weak positive expression (5). *In situ* hybridization for Epstein-Barr virus-encoded small RNA (EBER) yielded negative results in the tumor tissue (**Figure 2**).

**Figure 3:**
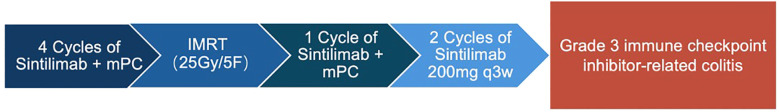
Timeline of postoperative treatment course and irAE.

After surgery, the patient received a chemotherapeutic regimen of micellar paclitaxel combined with cisplatin, along with concurrent immunotherapy. Based on the Du Bois formula, his body surface area was calculated as 1.81 m². The treatment plan consisted of 5 cycles administered every 21 days, with the following specific medication protocol: micellar paclitaxel 165 mg/m² administered on day 1, cisplatin 16.57 mg/m² administered on days 1-3, combined with the anti-PD-1 monoclonal antibody sintilimab 200 mg administered on day 1 (**Figure 3**).

**Figure 4:**
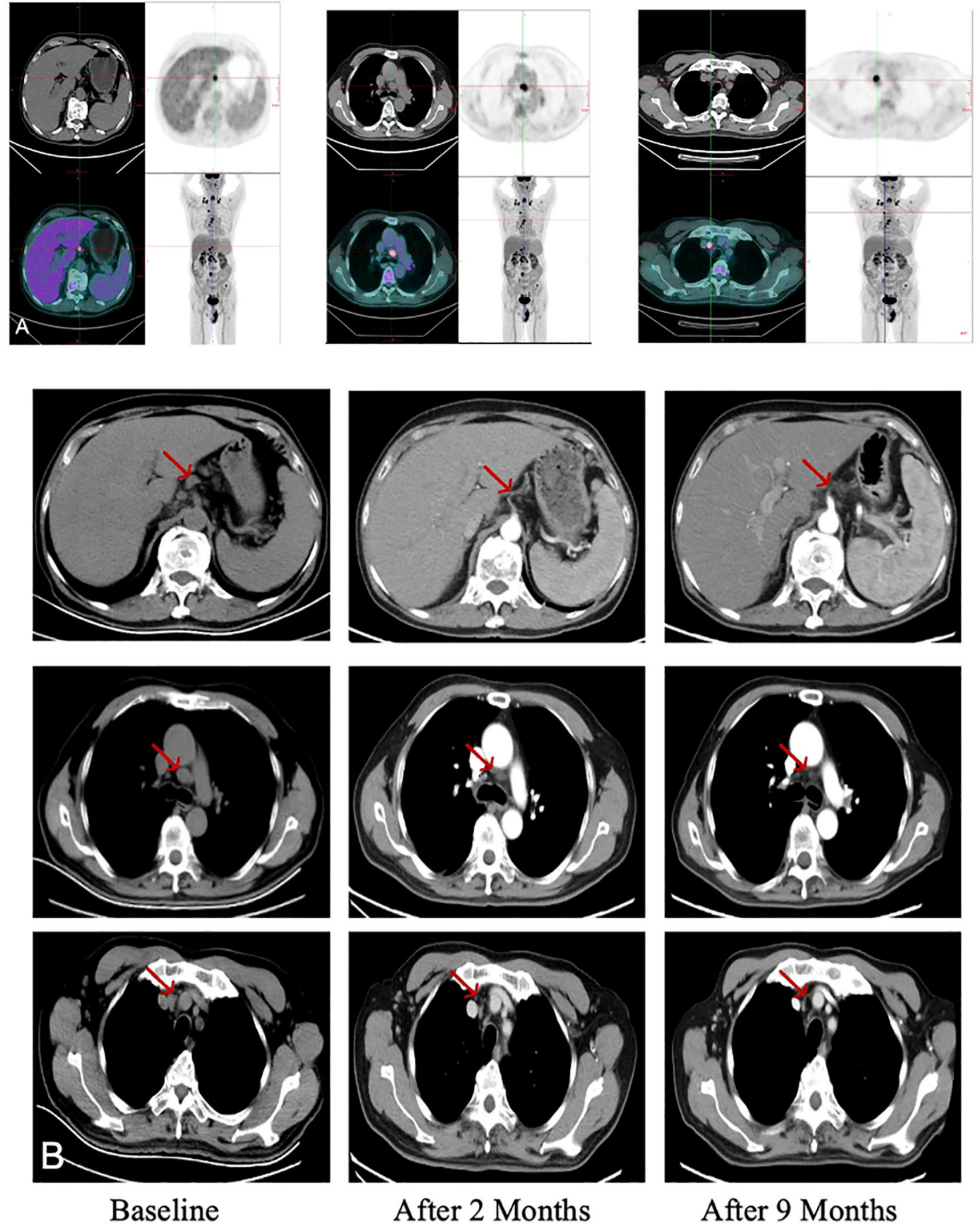
Comparative imaging demonstrates the evolution of metastatic lymph nodes. **(A)** Initial 18F-FDG PET-CT reveals extensive hypermetabolic lymphadenopathy in the mediastinal, gastrohepatic, and porta hepatis regions. **(B)** Computed tomography (CT) scans at baseline (pre-treatment) and at two and nine months post-treatment show serial regression of lymph node lesions. Red arrows indicate the regressing lesions at the specified sites.

**Figure 5:**
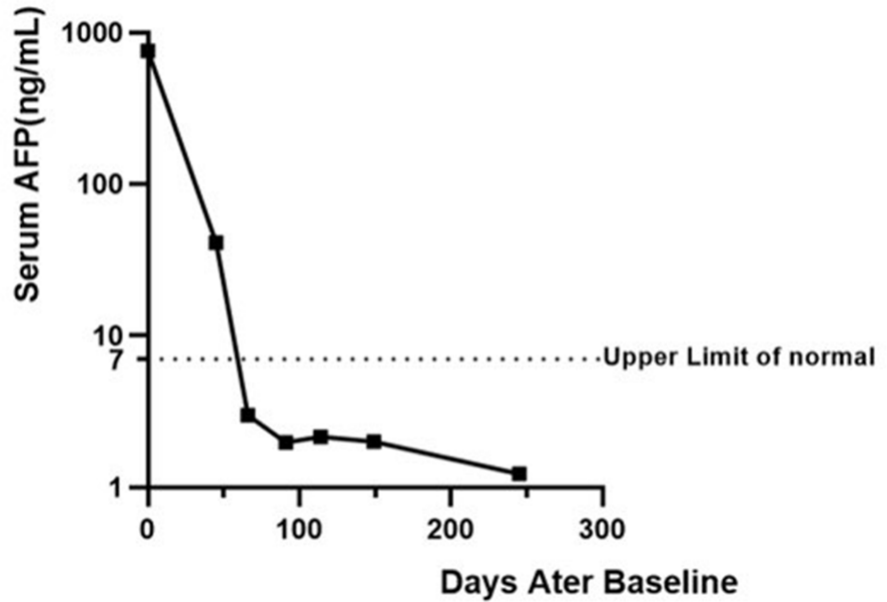
Dynamic changes of serum alpha-fetoprotein (AFP) levels following treatment. The Y-axis is displayed on a logarithmic scale to clearly depict both the initial rapid decline and subsequent stabilization at low levels. The dashed horizontal line indicates the upper limit of normal range (7 ng/mL). Baseline measurements were performed before the initiation of therapy.

Clinical efficacy was evaluated at intervals of every two treatment cycles. Given the patient’s prior partial hepatectomy, response was evaluated by monitoring the hypermetabolic lymph nodes on 18-FDG PET/CT (**Figure 4A**). Compared to baseline, imaging evaluations at two and nine months post-treatment demonstrated a reduction in the size of the lymph node lesions (**Figure 4B**). Notably, significant regression was observed in lesions located at the hepatogastric space, the right tracheobronchial area, and the retrosternal lymph nodes (indicated by red arrows in **Figure 4B**). Simultaneously, the patient’s serum alpha-fetoprotein (AFP) level normalized after two months following the initiation of treatment, decreasing to within the normal range (0–7 ng/mL) from a pretreatment level of 757.8 ng/mL, which correlated significantly with the imaging response (**Figure 5**).

After completing the fifth chemotherapy cycle, the patient developed treatment-related fatigue and decreased appetite. Satisfied with the current treatment outcome and prioritizing quality of life over prolonged survival, the patient expressed a preference for a less aggressive approach and declined further chemotherapy. Considering the patient’s preference, subsequent treatment was transitioned to an immunotherapy maintenance phase (sintilimab 200 mg every 3 weeks), planned to last for 2 years. As of the writing of this report, ongoing imaging surveillance continues to show partial response, with no evidence of new lesions or disease progression.

Notably, during the maintenance immunotherapy phase, the patient developed hematochezia and diarrhea after the second cycle of this phase. Laboratory results indicated positive fecal occult blood, normal procalcitonin (PCT), but significantly elevated IL-5 (5.34 pg/mL) and IL-6 (27.96 pg/mL). According to Common Terminology Criteria for Adverse Events (CTCAE) version 5.0, the condition was diagnosed as grade 3 immune checkpoint inhibitor-related colitis. The patient achieved substantial clinical improvement after 3 days of IV methylprednisolone (40 mg). After dose reduction to 20 mg IV for one day, therapy was converted to equivalent oral methylprednisolone maintenance at discharge, with completion of a standardized glucocorticoid tapering regimen under outpatient supervision. However, one month after the initial resolution of symptoms, hematochezia and diarrhea recurred. After excluding infectious or other potential causes, immune-related colitis was again considered the likely diagnosis. The patient was treated with infliximab (300 mg) in combination with corticosteroids and other supportive therapies, which led to a significant improvement in symptoms. The positive response to treatment supported the diagnosis of immune-related colitis. Regarding subsequent treatment options, we recommend multidisciplinary discussion to determine the optimal therapeutic strategy.

## 3 Discussion

To the best of our knowledge, this is the first report on grade 3 immune-mediated colitis in a LELC patient. For the first time, we explored the combination of micellar paclitaxel plus Cisplatin chemotherapy with immunotherapy in patients with LEL-HCC.

Lymphoepithelioma-like carcinoma (LELC), also known as lymphoepithelial carcinoma (LEC),is a malignant tumor characterized by undifferentiated epithelial cells with a prominent lymphocytic infiltrate, histologically resembling undifferentiated nasopharyngeal carcinoma (6, 7).This disease was initially discovered in the nasopharynx, with subsequently reported in multiple other anatomical sites, including the lungs, liver, digestive tract, bladder, and prostate (8–12). It is noteworthy that the EBV positivity rate, along with its epidemiological and clinical characteristics in LELC varies significantly across different anatomical sites. This variation may be influenced by factors such as the anatomical location of the tumor, geographical distribution, and genetic background (13). Hepatic LELC can be further classified into LEL-HCC and lymphoepithelioma-like cholangiocarcinoma (LEL-CC) (6). In 1998, Japanese researchers initially defined LELC histologically by the presence of more than 100 tumor-infiltrating lymphocytes per 10 high-power microscopic fields (14). The 2004 World Health Organization (WHO) Classification of Thoracic Tumors classified LELC as a subtype of large cell carcinoma. Since being designated as a distinct hepatocellular carcinoma subtype by the WHO in 2010, globally reported cases of this disease have progressively increased (15, 16). Accordingly, LELC was categorized under “other and unclassified carcinomas” in the 2015 WHO classification of thoracic tumors (17). The fifth edition of the WHO Classification of Tumours of the Digestive System (2019) categorizes hepatocellular carcinoma into eight subtypes, introducing “lymphocyte-rich hepatocellular carcinoma” as a new distinct entity. It is histologically defined by the feature that “on H&E staining, lymphocytes outnumber tumor cells in most fields,” and is described as not associated with Epstein-Barr virus (EBV) (4).Most recently, the 2021 WHO classification renamed it LEC and categorized it within the spectrum of squamous cell carcinomas (4).According to the global literature, 110 cases of hepatic LELC had been reported worldwide by 2022. The case distribution included: LEL-HCC (71 cases, 64.5%), LEL-CC (38 cases, 34.5%), and mixed LEL-HCC/LEL-CC (1 case, 0.9%) (18). Statistical analysis of incidence rates reveals that LEL-HCC primarily occurs in Asian males (64%), in contrast to LEL-CC which shows female predominance (66.7%). Geographically, 92% of cases have been reported in Asian populations. Among patients with LEL-HCC, the vast majority (88.4%) presented with solitary tumors. Approximately half of the cases demonstrated elevated AFP levels. Regarding tumor staging, the Barcelona Clinic Liver Cancer classification was predominantly stage 0 or A (6). Compared with conventional HCC, hepatic LELC demonstrates more favorable prognosis. Patients with LEL-HCC achieve a 5-year overall survival rate of up to 94.1% (19).

The negative EBV serology and EBER *in situ* hybridization results in this case further support the notion that LEL-HCC lacks a consistent association with EBV infection. The association between EBV and LELC demonstrates significant anatomic site heterogeneity. For instance, correlations with EBV infection have been established in LELCs of the lung (20, 21), stomach (22), and head and neck regions (23). This process may be driven by EBV-induced epigenetic modifications that facilitate the process of tumor development (24). While the 2010 WHO classification of digestive tumors acknowledged that not all LELCs are EBV-positive, the 2019 edition further clarified that the lymphocyte-rich subtype is not EBV-related. Significant differences in EBV positivity rates exist among the histological subtypes of hepatic LELC, with LEL-CC reaching 76.5% compared to merely 2.3% (1/42) in LEL-HCC (15). This discrepancy suggests that EBV infection is not a primary driving factor in LEL-HCC pathogenesis. It is noteworthy that the EBV infection rate exhibits geographical variation, suggesting potential complex interactions among viral, genetic, and environmental factors (22). Future studies are warranted to elucidate the differences in molecular pathways and tumor heterogeneity between EBV-positive and EBV-negative LELC cases, which will ultimately reveal the potential role of EBV in LELC pathogenesis.

Beyond EBV, hepatitis virus infections are also relatively common in LEL-HCC. Epidemiological data indicate that 42.6% of LEL-HCC patients are infected with hepatitis B virus (HBV), while 33.3% have concurrent hepatitis C virus (HCV) infection (15).However, there is currently no direct evidence to establish a causal relationship between HBV or HCV infection and the pathogenesis of LEL-HCC. This case presented with a specific serological pattern: HBsAb (–), HBeAb (–), and HBcAb(+). Such laboratory results reinforce the notion that the link between LEL-HCC and HBV/HCV infection is not consistently supported. We acknowledge that baseline HBV DNA quantification was not performed in this patient, which represents a limitation of this study. Nevertheless, the patient maintained normal liver function throughout treatment with close monitoring. Future investigations should integrate multi-omics analyses, functional virological studies, and clinical correlation research to elucidate the roles of these viral factors in the pathogenesis and progression of LEL-HCC.

In this case, given that two needle biopsies in this case were non-diagnostic, the limitations of needle biopsies are clearly demonstrated, thereby underscoring the necessity for surgical resection to achieve accurate diagnosis for such rare tumors. The diagnosis of LELC is primarily based on its characteristic histopathological features, which include poorly differentiated or undifferentiated atypical tumor cells accompanied by a prominent lymphocytic infiltrate. The tumor-infiltrating lymphocytes (TILs) are predominantly composed of cytotoxic T lymphocytes (CTLs) (19). According to the 2019 WHO classification, a key diagnostic feature of LELC is the quantitative histological threshold requiring lymphocytes to outnumber tumor cells in the majority of Hematoxylin and Eosin-stained microscopic fields (25).This criterion provides critical differentiation from morphologically similar tumors. Hepatic LELC can be further categorized into LEL-HCC and LEL-CC. Their discrimination relies on both histological morphology and immunophenotypic characteristics. The diagnostic principles for differentiating these two subtypes are analogous to those used in distinguishing conventional HCC from intrahepatic cholangiocarcinoma (ICC). Immunohistochemistry (IHC) plays a pivotal role in this subtyping: markers such as AFP, Hepatocyte, and GPC3 are commonly used to indicate hepatocellular differentiation, whereas CK19 and CK7 are more frequently expressed in tumors with cholangiocytic differentiation (5, 6, 26). Prior to the 2019 WHO classification, LEL-HCC was distinguished from lymphocyte-rich hepatocellular carcinoma by its typically poor differentiation and only focal or weak expression of hepatocellular markers such as Hepatocyte and Arg-1. However, it has now been reclassified as a subtype of lymphocyte-rich HCC (27). This diagnostic difficulty, which stems from the unique pathological nature of LELC, presents a significant challenge in clinical practice.

The patient reported in this case had an initial AFP level of 757.8 ng/mL at diagnosis, which was significantly higher than normal levels. After two months following the initiation of treatment, the AFP level showed a marked decline, indicating both treatment efficacy and the value of this serum tumor marker in the management of LEL-HCC. These features collectively underscore the unique characteristics as well as the similarities between LEL-HCC and conventional HCC. Regarding AFP levels, it has been reported that approximately half of LEL-HCC patients exhibit elevated AFP (6).Since its discovery in 1964 as a biomarker associated with liver cancer, AFP has been widely used for screening high-risk populations for HCC, evaluating treatment efficacy, and monitoring recurrence (28). Currently, circulating tumor DNA (ctDNA) testing for minimal residual disease (MRD) has been progressively integrated into clinical practice for colorectal cancer, particularly in post-operative recurrence monitoring and adjuvant chemotherapy decision-making (29).In pulmonary LELC, serum neuron-specific enolase (NSE) concentration may serve as a predictive biomarker for treatment efficacy (30).In the field of hepatocellular carcinoma research, despite its limitations in both sensitivity and specificity, serum AFP level detection remains the only approved biomarker for MRD (31, 32).It is hoped that future basic and clinical studies on AFP in LEL- HCC will yield more effective tools for disease management.

Unlike previously reported cases of hepatic LELC demonstrating high FDG uptake in primary liver lesions (33, 34) this case first documents the concurrent presence of FDG hypometabolism in the primary hepatic LELC lesion alongside hypermetabolism in metastatic lymph nodes, suggesting the potential existence of unique metabolic heterogeneity in hepatic LELC. This phenomenon holds significant implications for clinical practice in imaging diagnosis and disease surveillance. The hypometabolism observed in primary lesions may lead to false-negative interpretations, while the hypermetabolic features of metastatic sites could offer a sensitive indicator for ¹^8^F-FDG PET/CT monitoring during follow-up. Future investigations with larger cohort studies are needed to verify whether this metabolic heterogeneity represents a distinctive phenotypic pattern specific to hepatic LELC.

Regarding the treatment selection, based on our review of previous cases in treating HCC and LELC of other organs, we formulated a therapeutic plan combining chemotherapy with immunotherapy for the patient. The patient presented with extensive metastases at initial diagnosis, a situation carrying a grave prognosis without active management. Thus, employing aggressive therapy to swiftly control the tumor was imperative. For patients with early-diagnosed LEL-HCC, radical surgical resection or liver transplantation remains the preferred treatment strategy (16). Due to limited resources, only a small number of patients with LEL-HCC have undergone liver transplantation (35). The post-transplant recurrence rate remains below 9.1%, with 5-year survival rates ranging from 94.1% to 100% (3, 36). Currently, there are no established authoritative treatment guidelines for locally advanced or metastatic LEL-HCC. Most treatment approaches involve chemotherapy-based comprehensive regimens combined with targeted therapy, radiotherapy, and immunotherapy, though no consensus has been reached regarding optimal treatment strategies.

In this case, informed by the partial squamous differentiation of LELC, we opted for a chemotherapy regimen comprising a platinum drug combined with the modern micellar formulation of paclitaxel. In LELC occurring at various sites such as breast and head-neck regions, platinum-based chemotherapy regimens combined with antimetabolites (5-fluorouracil, pemetrexed) or anti-tubulin agents (paclitaxel) have become the mainstream treatment strategy (21, 37, 38). In the first-line treatment of advanced pulmonary LELC, platinum-based regimens combined with taxanes (TP regimen) or gemcitabine (GP regimen) demonstrate significantly higher clinical benefit rates compared to platinum-based therapy combined with pemetrexed (AP regimen) (21). In LEL-HCC, platinum-based chemotherapy regimens centered on 5-fluorouracil (5-FU) have also been supported by case reports in the treatment of this malignancy (38, 39). Attempts have also been made with oral targeted agents such as sorafenib (2) or lenvatinib (18, 40–43) with reported applications also existing in hepatic LELC cases (38).

Given the molecular and pathological profile of this LELC, coupled with clinical studies and reports demonstrating the benefit of ICIs in LELC of the lung, stomach, and other organs, we deemed it justified to incorporate sintilimab immunotherapy into the treatment regimen for this patient. At the cellular and molecular level, the immunogenic microenvironment exhibited by LELC provides a theoretical foundation for the application of ICI. The most prominent pathological feature of LELC is the substantial inflammatory cell infiltration within tumor tissue. Its tumor microenvironment (TME) is primarily composed of CD4+ and CD8+ cytotoxic T cells, accompanied by scattered germinal center structures containing B cells. It has been reported that CCL20 expression levels were significantly higher in LR-HCC than in non-LR-HCC (P< 0.01). Among tumor-infiltrating lymphocytes, the number of CD3-positive cells was significantly greater than that of CD20-positive cells (P< 0.0001), with the majority being CD8-positive T cells (44). Studies have shown that in lymphocyte-rich hepatocellular carcinoma (LR-HCC), the number of CD3+ T cells significantly exceeds that of CD20+ B cells (ratio 1.5:1, P< 0.001), and CD8+ cytotoxic T cells are more frequently detected than Foxp3+ regulatory T cells (ratio 2.4:1, P< 0.001) (45). PD-L1 is expressed on both tumor cells and immune cells in LR-HCC, suggesting that this subtype possesses distinct immunogenic characteristics (46).This distinctive immune cell composition pattern suggests significant alterations in the local tumor immune microenvironment (47). Although LEL-HCC shows no significant differences from conventional HCC in molecular characteristics including microsatellite instability (MSI) status, BRAF mutations, and CpG island hypermethylation, genomic studies indicate that LEL-HCC possesses a unique immunogenetic background (19). For instance, the chromosomal region 11q13.3, which contains genes such as CCND1 and FGF19, demonstrates specific alterations in hepatic LELC. This genomic region is known to be associated with programmed death-ligand 1 (PD-L1) expression and immune checkpoint regulation (48). In the tumor immune microenvironment of Epstein-Barr virus-associated lymphoepithelioma-like cholangiocarcinoma, genetic mutations such as BARD1, CD19, CD79B, and TNFAIP3 have been identified, along with characteristic high expression of PD-L1 in tumor-infiltrating immune cells. These molecular findings provide support for the potential application of immune checkpoint inhibitors in the treatment of this tumor type (47).

Building upon these distinctive immune microenvironment features, ICI are gaining increasing attention in the clinical management of LELC (10, 47, 49, 50). In pulmonary LELC, the PD-L1 positivity rate reaches as high as 74.3%, and PD-1/PD-L1 inhibitors have demonstrated significant efficacy in patients with this malignancy (50) (51). In gastric LELC, upregulated PD-L1 expression has been consistently observed, and this tumor type also demonstrates sensitivity to immunotherapy (52). Currently, the clinical application of ICI in hepatic LELC is supported only by limited case reports. Successful cases demonstrating response to nivolumab (38)and toripalimab (18) in advanced LEL-HCC have confirmed the potential of ICI therapy for hepatic LELC.

Notably, six months after initiating immunotherapy, corresponding to two cycles of maintenance therapy, the patient developed gastrointestinal adverse effects including diarrhea and hematochezia. After excluding infectious causes, the patient was diagnosed with grade 3 immune-related colitis according to CTCAE v5.0 criteria, which showed significant improvement following glucocorticoid treatment. Laboratory tests revealed serum anti-Ro52 antibody positivity at initial diagnosis, with all other rheumatologic autoantibodies being negative, along with persistently elevated levels of IL-5 and IL-6. Although anti-Ro52 antibody is a member of the anti-Ro/SSA antibody family and serves as a serological marker for Sjögren’s syndrome and systemic lupus erythematosus, its detection in non-autoimmune diseases has raised questions about its specificity, and its clinical significance remains controversial (53, 54). Research indicates that autoimmune diseases and their family history may increase the risk of irAEs in patients receiving ICI therapy (55). Furthermore, cytokines play a significant mediating role in the pathogenesis of irAEs (56–58). Whether this patient possesses a molecular biological basis for susceptibility to irAEs requires further investigation at the molecular level for confirmation.

Current clinical reports in the literature regarding irAEs in LELC patients receiving immunotherapy primarily focus on grade 1–2 mild adverse reactions, such as skin rash, with no documented cases of grade 3 or higher moderate-to-severe irAEs reported to date (59). This case represents the first report of grade 3 immune-mediated colitis in a LELC patient, providing important clinical reference for safety evaluation and clinical management of immunotherapy in hepatic LELC. Current evidence suggests that among patients who discontinued ICI therapy due to irAEs and were subsequently rechallenged with the same agent, 28.8% experienced recurrence of the same irAE (60). For carefully selected patients, ICI therapy may be reconsidered following the establishment of a comprehensive toxicity monitoring system and standardized treatment protocols (61). However, when patients develop moderate-to-severe irAEs while demonstrating confirmed immunotherapy benefits, the formulation of subsequent treatment strategies remains a major clinical dilemma. These decisions include whether to permanently discontinue treatment, adjust ICI dosage, switch to alternative immunotherapeutic agents for rechallenge, or transition to low-dose oral chemotherapy regimens. This represents a critical scientific problem requiring urgent resolution in LELC immunotherapy and demands more high-quality clinical case reports and further investigations to establish evidence-based medical guidance.

## 4 Conclusion

We describe a patient with advanced LEL-HCC who attained sustained partial response and maintained disease control for 8 months following a combined therapy protocol involving immune checkpoint inhibition, chemotherapy, and irradiation. This outcome supports the potential of micellar paclitaxel and cisplatin-based chemotherapy combined with ICI and radiotherapy as a viable strategy for unresectable metastatic LEL-HCC. The favorable clinical course may be attributed to the distinct immune microenvironment features, including tumor-infiltrating lymphocytes. Notably, this is the first report of steroid-responsive immune-related colitis in a LEL-HCC patient, highlighting safety management during immunotherapy for this malignancy. Future multicenter studies are warranted to refine therapeutic approaches and elucidate immunopathological mechanisms of this rare hepatic neoplasm.

## Data Availability

The original contributions presented in the study are included in the article/supplementary material. Further inquiries can be directed to the corresponding author.
